# Quantitative Structure-Property Relationship (QSPR) Modeling of Drug-Loaded Polymeric Micelles via Genetic Function Approximation

**DOI:** 10.1371/journal.pone.0119575

**Published:** 2015-03-17

**Authors:** Wensheng Wu, Canyang Zhang, Wenjing Lin, Quan Chen, Xindong Guo, Yu Qian, Lijuan Zhang

**Affiliations:** 1 School of Chemistry and Chemical Engineering, South China University of Technology, Guangzhou, P. R. China; 2 School of Chemistry and Chemical Engineering, Zhaoqing University, Zhaoqing, P. R. China; Mathematical Institute, HUNGARY

## Abstract

Self-assembled nano-micelles of amphiphilic polymers represent a novel anticancer drug delivery system. However, their full clinical utilization remains challenging because the quantitative structure-property relationship (QSPR) between the polymer structure and the efficacy of micelles as a drug carrier is poorly understood. Here, we developed a series of QSPR models to account for the drug loading capacity of polymeric micelles using the genetic function approximation (GFA) algorithm. These models were further evaluated by internal and external validation and a *Y-randomization* test in terms of stability and generalization, yielding an optimization model that is applicable to an expanded materials regime. As confirmed by experimental data, the relationship between microstructure and drug loading capacity can be well-simulated, suggesting that our models are readily applicable to the quantitative evaluation of the drug-loading capacity of polymeric micelles. Our work may offer a pathway to the design of formulation experiments.

## Introduction

Cancer, as one of main diseases, will soon replace heart disease to become world's leading killer, seriously threatening human health [1]. Chemotherapy is an effective method for the treatment of cancer. However, the toxicity and side effects of anticancer drugs can be life threatening, and most of these drugs suffer from poor water solubility, have short half-lives, and can cause tumor cells to become drug resistant [2]. Extensive research is therefore being conducted on new formulations that might effectively improve the curative effect of anticancer drugs while minimizing or even eliminating their toxicity and side effects. One promising approach is to consider using a new drug delivery system to control the target delivery of drugs to the lesion [3,4]. Nano-micelles self-assembled from amphiphilic polymers represent a candidate system due to their many advantages, including small particle size, release behavior, improved drug solubility and the potential for both passive and active targeting ability [5–9]. Until now, many polymers have been extensively investigated for use in constructing micelles for drug delivery [10–16]. Linear block polymers were studied first due to their simple structures. Although some of these polymers reached the clinical evaluation stage, these polymers exhibit limited stability and burst release due to the dynamic nature of self-assembly [17–20]. Thus, high-grafting-density comb polymers and dendritic polymers have been developed to improve the stability and release performance of the drug-loaded micelle system [21–23]. According to the in vitro experiments, the rates of the drug release from the polymeric micelles were obviously different at different pH conditions. Specifically, the drug release rate at weakly acidic circumstance (around tumor site) was much higher than that at normal condition, indicating that the drug-loaded polymeric micelles exhibited pH-responsive. With the rapid development of controllable active polymerization, many methods, especially atom transfer radical polymerization (ATRP) and ring opening polymerization (ROP), have been used to control the polymeric composition, topological structure and to prepare functional polymers [9]. However, to obtain an appropriate drug delivery system, extensive experimentation is required; such experiments are not only financially costly but also are highly time-consuming. Thus far, studies on the structure-performance relationship of micelles have been confined to qualitative studies, including molecular simulation and mesoscopic simulation methods, which only describe the chemical morphology, microstructure and mesoscopic phase separation of polymers or colloids [24–26]. Very few studies of the quantitative relationships involved have been reported. Due to lack of information on the QSPR, it is difficult to fundamentally understand the nature of the interactions between the structure of polymer molecules and the drug-loading and drug-release performances. Herein, the QSPR is the major issue addressed.

The GFA algorithm offers a new approach to developing structure-property models [27–31]. QSPR models can be created automatically by combining the use of a genetic algorithm with statistical modeling tools. In this method, a GFA is used to solve function approximation. This algorithm is initially developed from two apparently disparate algorithms: Holland’s genetic algorithm and Friedman’s multivariate adaptive regression splines algorithm [32,33]. The GFA algorithm makes use of a population of many models and tests only the final, fully constructed model rather than generating a single model. The models are scored using Friedman's “lack of fit” (LOF) measure as the evaluation function [34,35]. This algorithm has been successfully applied to generate a variety of QSPR models.

QSPR studies are very useful for obtaining in-depth insights into structure-property relationships. In this article, the QSPR is studied *via* the GFA technique based on a series of four/six-arm star polymer structures and the data regarding the DOX-loading capacities of the micelles. The descriptors in the QSPR are also selected using a GFA algorithm. Both cross validations of multiple linear regression (MLR) and the leave-one-out (LOO) method were used to build and evaluate the QSPR models. The characteristics of these models, including fitting ability, predictive ability, stability and generalization ability, are evaluated by internal and external validation and the *Y-randomization* test. We also define the applicability domain of the optimization model. The results obtained provide important guidance for the design and synthesis of the desired polymers.

## Materials and Methods

### Experimental data and data splitting

**Fig 1:**
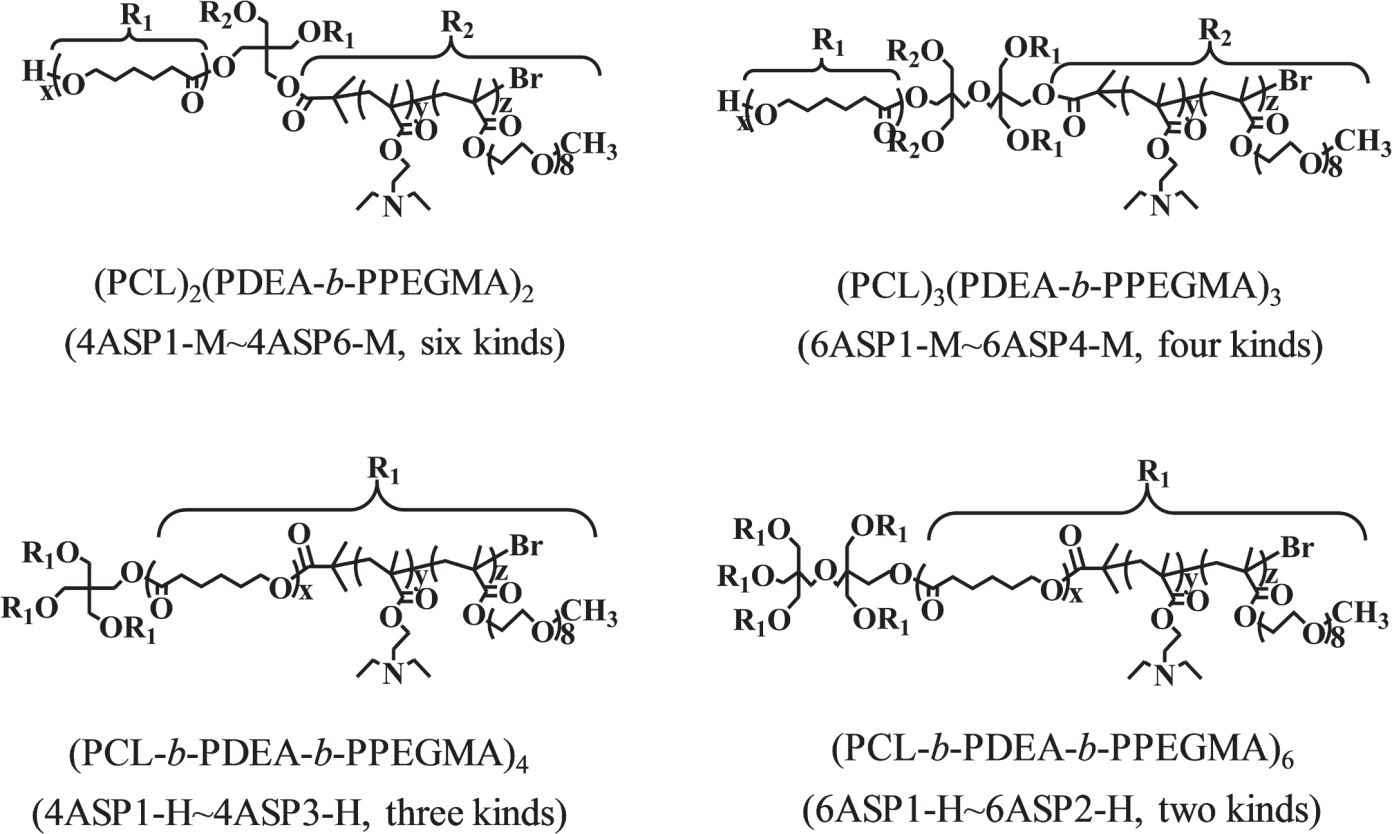
The structures of four- and six-armed polymers.

The research system used was star polymers, and the drug-loading capacities (LC, % w/w) of micelles self-assembled from these star polymers were used as the performance measure. In this study, 15 kinds of star polymers were used, including six kinds of four-miktoarm star polymers (PCL)_2_(PDEA-*b*-PPEGMA)_2_, four kinds of six-miktoarm star polymers (PCL)_3_(PDEA-*b*-PPEGMA)_3_, three kinds of four-homoarm star polymers (PCL-*b*-PDEA-*b*-PPEGMA)_4_, and two kinds of six-homoarm star polymers (PCL-*b*-PDEA-*b*-PPEGMA)_6_. Fifteen kinds of designed polymers were synthesized in our laboratory. Drug-loaded micelles were prepared using two DOX/polymer ratios (10/40 and 20/40 (mg/mg)), resulting in 30 data LC datasets (LC datasets were transformed using natural logarithms (ln) to better conform to the normal distribution), as shown in S1 Table [36–38]. All LC data were measured in aqueous solution at pH 7.4 and room temperature. All details are shown in Fig. 1 and S1 Table.

To build and validate a QSPR model of good general ability, the original dataset was divided into a training set and a test set using the Duplex method which was an effective way to select representative training and test sets [39]. Briefly, Euclidean distances were calculated between each pair of samples as follows: (1) The two samples that are furthest away from each other are selected for the training set. (2) From the remaining samples, the two samples that are furthest away from each other are included in the test set. (3) The remaining point that is furthest away from the two points in step (1) is included in training set, and the one furthest away from the two points in step (2) is included in the test set. Finally, the datasets were split into the training set (containing 22 data) and the test set (containing 8 data, but not less than five) [40]. See S1 Table for a detailed list.

### Computational details regarding descriptors

Total fifty-two kinds of molecular descriptors (including physical and chemical descriptors, fragment counts, topological, spatial and energy descriptors, atomic volumes and surfaces, and atomistic descriptors which are suitable for describing the relationship between polymer molecular structure and micellar property) could been computed using the QSAR module of Materials Studio 5.0 (Accelrys Inc.) [41] and used as candidates, as shown in S2 Table. To obtain the lowest energy conformation of the polymer molecules, the geometric structures of all polymer molecules were constructed and optimized using the following three steps. First, the initial polymer structure was constructed in the Materials visualizer. Then, the polymer structures were minimized in the Discover Module by setting the optimization method to smart minimizer, the force field type to compass, the convergence level to ultra-fine, the non-bond methods to van der Waals and Coulomb interactions, and the summation method to atom-based. Finally, the structures were optimized using molecular dynamics simulation for the NVE and NVT ensembles in turn, and the temperature was set to 298K; the number of steps was set to 10,000. Then, values of 52 descriptors were calculated using the QSAR and Forcite Modules of Materials Studio 5.0 based on the polymer structure that was optimized as described above. These descriptor values were separated according to the quality ratios of the drug/polymer. Constant terms, near-constant values and pairwise-correlated descriptors (one of any two descriptors with a correlation coefficient greater than 0.99) were excluded in a pre-reduction step. The remaining 36 descriptors and their detailed values for the 22 polymers, showing in S3 and S4 Tables, were selected and calculated for the following research.

### Model development

In this study, some methods were used to develop and evaluate a reliable model. GFA-MLR technique, derived from an analogy with the evolution of DNA [42], was used to generate a series of models. In GFA algorithm, an individual or model was represented as one-dimensional string of bits. It was a distinctive characteristic of GFA that it could create a population of models rather than a single model. GFA algorithm, selecting the basis functions genetically, developed better models than those made using stepwise regression methods.

And then, the models were estimated using the “lack of fit” (LOF), which was measured using a slight variation of the original Friedman formula [34,35], so that best model received the best fitness score. The LOF was determined by the following revised equation in Materials Studio 5.0:

$$LOF=\frac{SSE}{n{\left[1-\lambda \left(\frac{c+dp}{n}\right)\right]}^{2}}$$ (1)

where, *SSE* is the sum of squares of the errors, *c* is the number of terms in the model (other than the constant term), and *d* is a scaled smoothing parameter, which is used to evaluate the Friedman LOF statistic. Larger values of the smoothness parameter cause larger models to be penalized to a greater degree. *p* is the total number of descriptors contained in all model terms (ignoring the constant term), *n* is the total number of samples in the training set, and *λ* is a safety factor, which was assigned the value 0.99 to ensure that the denominator of the expression would never become zero.

The applicability domain for the best QSPR model should be defined [43]. In this work, we have applied the leverage approach to investigate the domain of applicability of polymers [44]. It is a great benefit of this approach that can draw the Williams plot (a plot of standardized predicted residuals versus leverage values) to observe the applicability domain of a QSPR model.

### Model validation

The fitting ability, stability, reliability and predictive ability of the developed models were evaluated by some validation [43–46], including internal and external validation parameters.

### Internal validation parameters

*R* ^*2*^: *R* ^*2*^ (the square of the correlation coefficient) describes the fraction of the total variation attributed to the model. The closer the value of *R* ^*2*^ is to 1.0, the better the regression equation explains the *Y* variable. *R* ^*2*^ is the most commonly used internal validation indicator and is expressed as follows:

$$R^{2}=1-\frac{\sum {\left(Y_{obs}-Y_{pred}\right)}^{2}}{\sum {\left(Y_{obs}-{\bar{Y}}_{training}\right)}^{2}}$$ (2)

where, $Y_{obs},Y_{pred},{\bar{Y}}_{training}$ are the experimental property, the predicted property and the mean experimental property of the samples in the training set, respectively.

*R* ^*2*^_*cv*_: *R* ^*2*^_*cv*_ (the cross-validated correlation coefficient) is derived from cross validation and is the cross-validated equivalent of *R* ^*2*^. In general, the developed models were subjected to internal validation using the LOO-cross-validation method. *R* ^*2*^_*cv*_ is expressed as follows:

$$R_{cv}^{2}=1-\frac{\sum {\left(Y_{obs}-Y_{pred}^{'}\right)}^{2}}{\sum {\left(Y_{obs}-{\bar{Y}}_{training}\right)}^{2}}$$ (3)

where, ***Y***_*obs*_ and ${\bar{Y}}_{training}$ have the same definitions as in Equation (2) above. $Y_{pred}^{'}$ is the LOO-predicted property of the samples in the training set. The closer this value is to 1.0, the better the predictive ability of the model [45].

*RMSE*: The root mean square error (*RMSE*) is dispersion degree of random error, presenting a more intuitive index of the fitting ability of the model [46], and is defined as follows:

$$RMSE=\sqrt{\frac{\sum_{i=1}^{n}{\left(Y_{obs}-Y_{pred}\right)}^{2}}{n}}$$ (4)

where, *i* represents sample *i* and *n* is the total number of samples in the training set. The lower the value of *RMSE* is, the better the predictive ability of the model.

*Y-randomization test*: The *Y-randomization* test is a statistical method that is widely used to test the reliability and robustness of a model [47]. The purpose of the *Y-randomization test* is to detect and quantify chance correlations between the dependent variable and the descriptor. In this method, a QSPR model is recalculated for randomly reordered responses, and the descriptor matrix remained unchanged. The obtained models should exhibit significantly lower values of *R* ^*2*^ or *R* ^*2*^_*cv*_ than the original model because the relationship between the structure and the response has been broken. *Y-randomization* is performed through response scrambling with maximum iterations of 500; then, the mean values of *R* ^*2*^ or *R* ^*2*^_*cv*_ are calculated. If either *R* ^2^ or *R* ^*2*^_*cv*_ is higher than 0.5, the original model is suspected to be relevant, and the reliability of the QSPR model is doubtful [48].

### External validation parameters

Internal validation is an essential step in QSPR model development. The desired internal validation results show that the model exhibits higher stability and prediction ability. However, no real prediction ability is shown for external samples. Therefore, the external predictive ability and extrapolation of the models should be evaluated.

*R* ^*2*^_*pred*_: *R* ^*2*^_*pred*_ is termed the predictive *R* ^2^ of a development model and is an important parameter that is used to test the external predictive ability of a QSPR model [48]. *R* ^*2*^_*pred*_ is defined as follows:

$$R_{pred}^{2}=1-\frac{\sum {\left(Y_{obs\left(test\right)}-Y_{pred\left(test\right)}\right)}^{2}}{\sum {\left(Y_{obs\left(test\right)}-{\bar{Y}}_{training}\right)}^{2}}$$ (5)

where, $Y_{obs}{}_{\left(test\right)},Y_{pred}{}_{\left(test\right)},{\bar{Y}}_{training}$ are the experimental property, the predicted property and the mean of the experimental property of the samples in the test set, respectively.

$\bar{r_{m}^{2}}$ and $\Delta r_{m}^{2}$: $\bar{r_{m}^{2}}$ and $\Delta r_{m}^{2}$ are developed to obtain a true predictive QSPR model based on the $r_{m}^{2}$ and $r^{'}{}_{m}^{2}$ parameters proposed by Roy et al [49]. $\bar{r_{m}^{2}}$, which has been found to be a better metric than the original $r_{m}^{2}$, is the average value of $r_{m}^{2}$ and $r^{'}{}_{m}^{2}$. $\Delta r_{m}^{2}$ is the absolute difference between $r_{m}^{2}$ and $r^{'}{}_{m}^{2}$. If the value of $\bar{r_{m}^{2}}$ is higher than 0.5, the value of $\Delta r_{m}^{2}$ should be lower than 0.2 [50]. In general, better models exhibit lower $\Delta r_{m}^{2}$ values. The equations for these parameters are shown in S5 Table.

### Applicability domain

After internal and external validation, it cannot be claimed that this model will provide reliable results for any unknown sample, even though all evaluation indexes prove that the model is stable and reliable and exhibits good generalization and predictive ability. In fact, each model has its own applicability domain. If the predictive value of the sample lies within this applicability domain, the value is reliable. On the contrary, the model remains unreliable [50]. Thus, it is necessary to define the applicability domain of the model before it is applied to simulate unknown samples.

The leverage approach is generally used to define applicability domain of a model, and the leverage approach requires the assumption that the datasets used follow the normal distribution [44]. This approach can quantify the applied range of the model, and the results can be presented using the Williams plot, in which the leverage values (or Hat values) and cross-validated standardized residuals are used as the abscissa and ordinate, respectively. In this plot, two horizontal lines and one vertical line delimit a safety zone. The critical leverage h* (the vertical line) is generally fixed at 3(*p* + 1)/*n* (where *n* is the number of training compounds and *p* is the number of variables used in the model, respectively). The leverage (*h*_*i*_) of every sample is defined as follows:

$$h_{i}=x_{i}{\left(X^{T}X\right)}^{-1}x_{i}^{T}(i=1,2\cdots ,m)$$ (6)

where, *x*_*i*_ is the descriptor row-vector of the query *i*th sample, *X* is the characteristic matrix of the training set, and *m* is the number of query samples. A higher leverage (*h*_*i*_ > h*, outside the safety zone) indicates that the predicted response is unreliable because it goes beyond reasonable extrapolation. If the cross-validated standardized residual of the sample is lower or higher than three standard deviation units (represented as the two horizontal lines), the sample remains outside the reasonable range. Only values that fall within this area are considered reliable.

## Results and Discussion

### Determination of the optimal descriptor number

**Fig 2:**
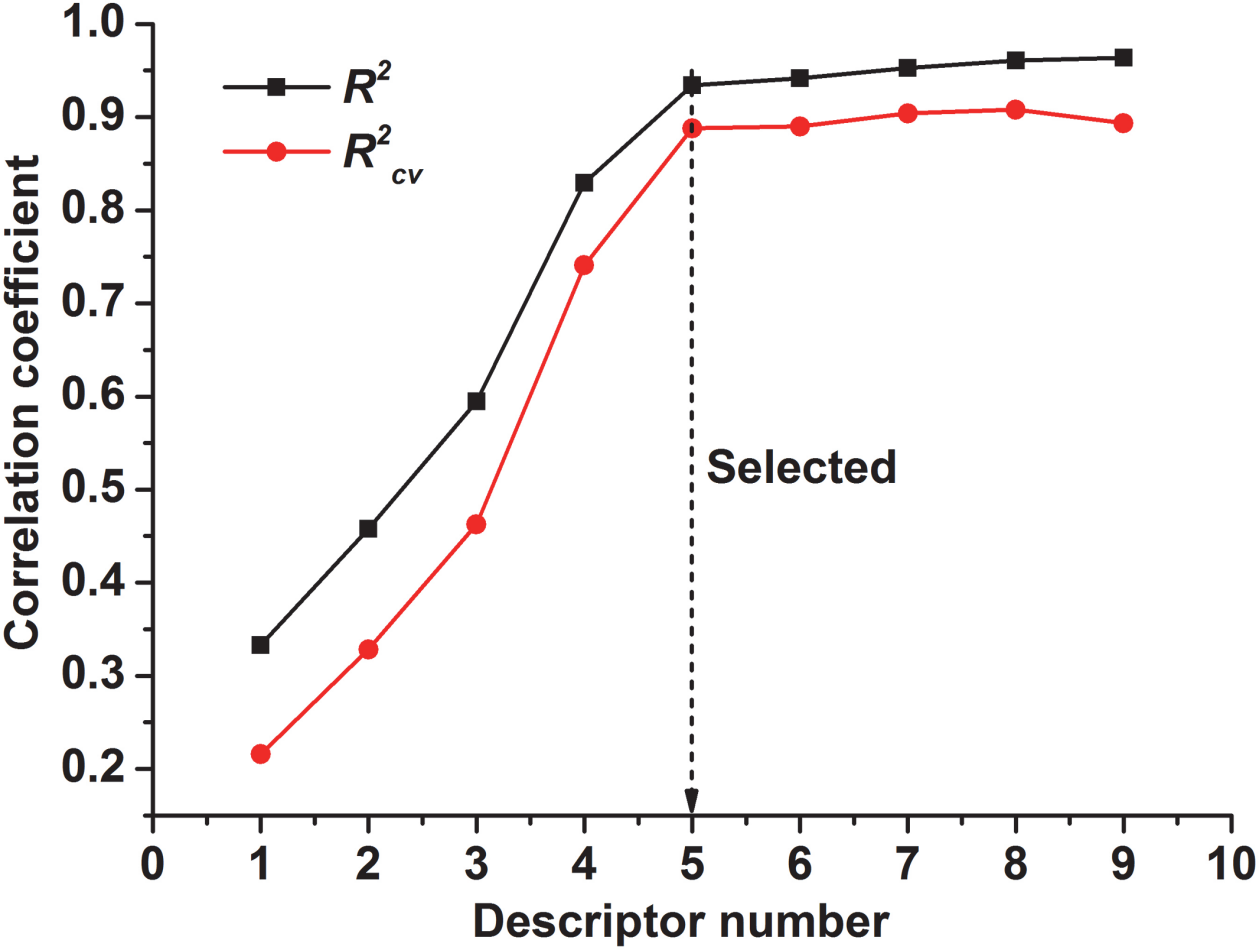
Dependence of the correlation coefficients *R^2^cv* and *R* ^*2*^ on the number of descriptors.

To select the descriptors that are most relevant to the ln(LC) of the polymeric micelles, 36 descriptors, which were calculated using Materials Studio, were used as inputs for the GFA algorithm. The optimal subset size was realized when further increases in the descriptor did not significantly improve model performance. Here, LOO-cross-validation was used to estimate the models. A plot of *R* ^*2*^_*cv*_ and *R* ^*2*^ against the number of selected descriptors is shown in Fig. 2 and indicates that the optimal model consists of five descriptors [39]. Moreover, *R* ^*2*^_*cv*_ is a key parameter regarding the predictive ability of the model. The closer the value of *R* ^*2*^_*cv*_ is to 1, the better prediction ability the model can deliver. For a good model, *R* ^*2*^_*cv*_ should be closer to *R* ^*2*^ (*R* ^*2*^_*cv*_ is usually lower). If *R* ^*2*^_*cv*_ is far smaller than *R* ^*2*^, then the possibility of data over-fitting in the regression model becomes significantly higher. When the number of descriptors is 5 (Fig. 2), *R* ^*2*^_*cv*_ fits *R* ^*2*^ most comfortably, indicating that the optimal subset size is five.

### Model building

To develop the optimization model, we included 22 samples in the training set. The number of descriptors in the regression equation was 5, and Population and Generation were set to 1,000 and 5,000, respectively. The number of top equations returned was 10 (starting from the tenth model, the value of *R* ^*2*^ is lower than 0.9). Mutation probability was 0.1, and the smoothing parameter was 0.5. The models were scored based on Friedman’s LOF. The statistical parameters of the ten models are shown in Table 1.

**Table 1:** Statistical parameters of the ten GFA-MLR models.

Model	*R* ^*2*^	*R* ^*2*^_*cv*_	*RMSE(a)*	*F*	*R* ^*2*^_*pred*_	*RMSE(b)*	*$\bar{r_{m(test)}^{2}}$*	*$\Delta r_{m(test)}^{2}$*
1	0.934	0.888	0.0675	45.31	0.811	0.1121	0.7500	0.0406
2	0.930	0.876	0.0683	42.52	0.810	0.1127	0.7563	0.0276
3	0.924	0.864	0.0724	38.96	0.766	0.1250	0.7096	0.0518
4	0.924	0.864	0.0726	38.77	0.809	0.1128	0.7451	0.1064
5	0.919	0.849	0.0749	36.22	0.811	0.1122	0.7545	0.0291
6	0.918	0.851	0.0754	35.72	0.804	0.1142	0.7392	0.1269
7	0.913	0.838	0.0774	33.66	0.773	0.1230	0.7091	0.0025
8	0.913	0.837	0.0774	33.66	0.808	0.1132	0.7513	0.0404
9	0.909	0.829	0.0794	31.85	0.773	0.1230	0.7073	0.0102
10	0.883	0.779	0.0899	24.16	0.745	0.1302	0.6187	0.1872

*RMSE (a)*: root mean square error of the training set; *RMSE (b)*: root mean square error of the test set. *F*: The *F* test is a standard statistical test for the equality of the variances of two populations having normal distributions.

**Fig 3:**
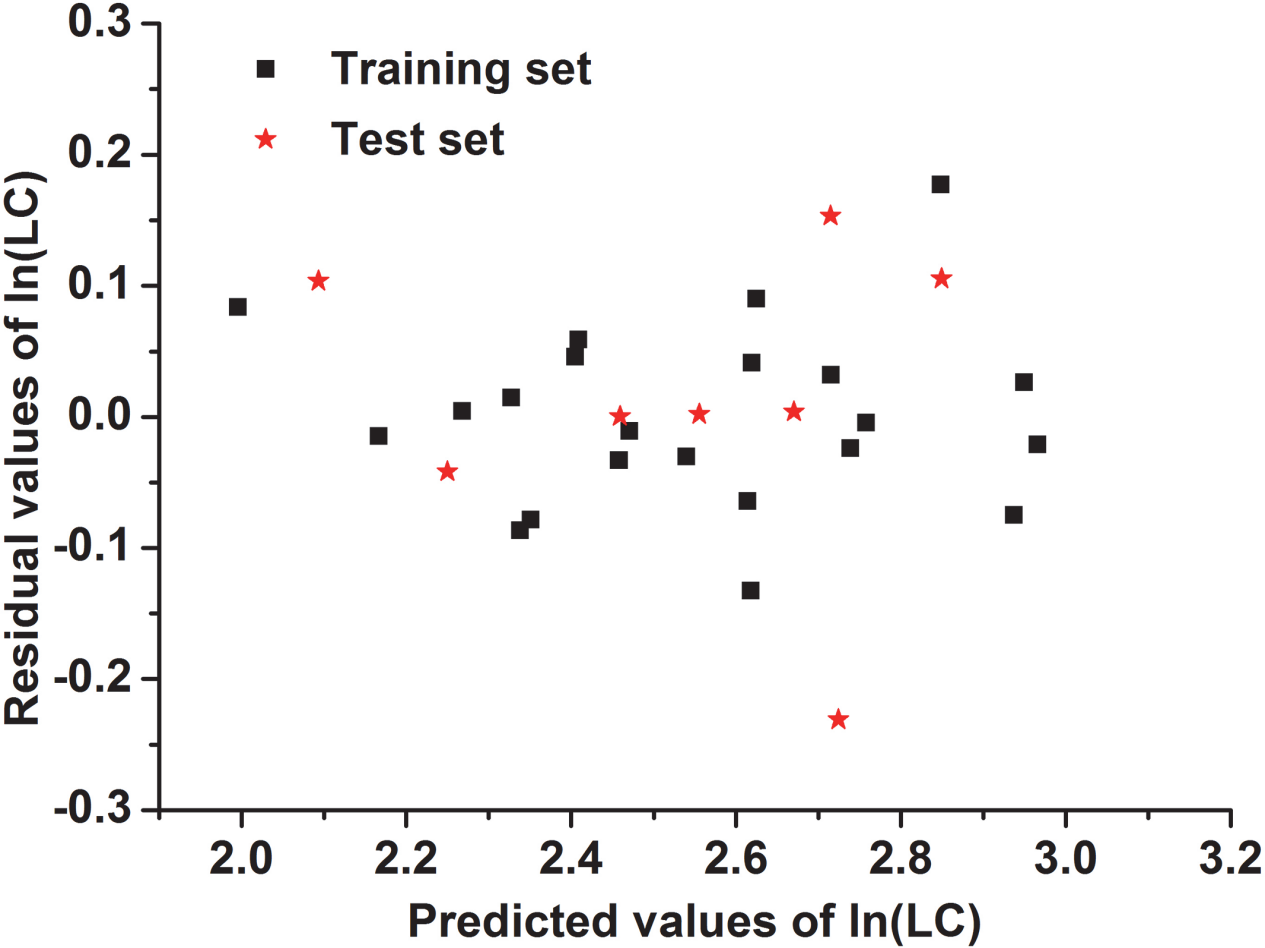
A scatter diagram of residuals for Model 1.

*R* ^*2*^, *R* ^*2*^_*cv*_ and *F* for Model 1 were 0.934, 0.888 and 45.31, respectively, and these values are the largest found among the ten models. Moreover, *RMSE(a)* was the smallest among the models, and the difference between *R* ^*2*^ and *R* ^*2*^_*cv*_ was the lowest. These results suggest that Model 1 exhibits the best fitting ability and the best internal predictive ability. To examine the stability of Model 1, the *Y-randomization* test was conducted, and a residual scatter diagram was plotted. After repeating the *Y-randomization* test more than 500 times, the mean values of *R* ^*2*^ and *R* ^*2*^_*cv*_ became 0.043 and 0.005, respectively, much lower than 0.5, indicating that Model 1 is more stable, and there is no “chance correlation” phenomenon occurring for Model 1. As seen in Fig. 3, the points are distributed irregularly and randomly, proving that Model 1 is more stable.

Regarding the external validation parameters for Model 1 (Table 1), *R* ^*2*^_*pred*_ is the highest (0.811) and *RMSE(b)* is the lowest (0.1121) among the models. The $\bar{r_{m(test)}^{2}}$ (0.75) of Model 1 is higher than 0.5, and the $\Delta r_{m(test)}^{2}$ (0.04) is much lower than 0.2. These results show that Model 1 exhibits slightly higher external prediction ability than the other models.

**Fig 4:**
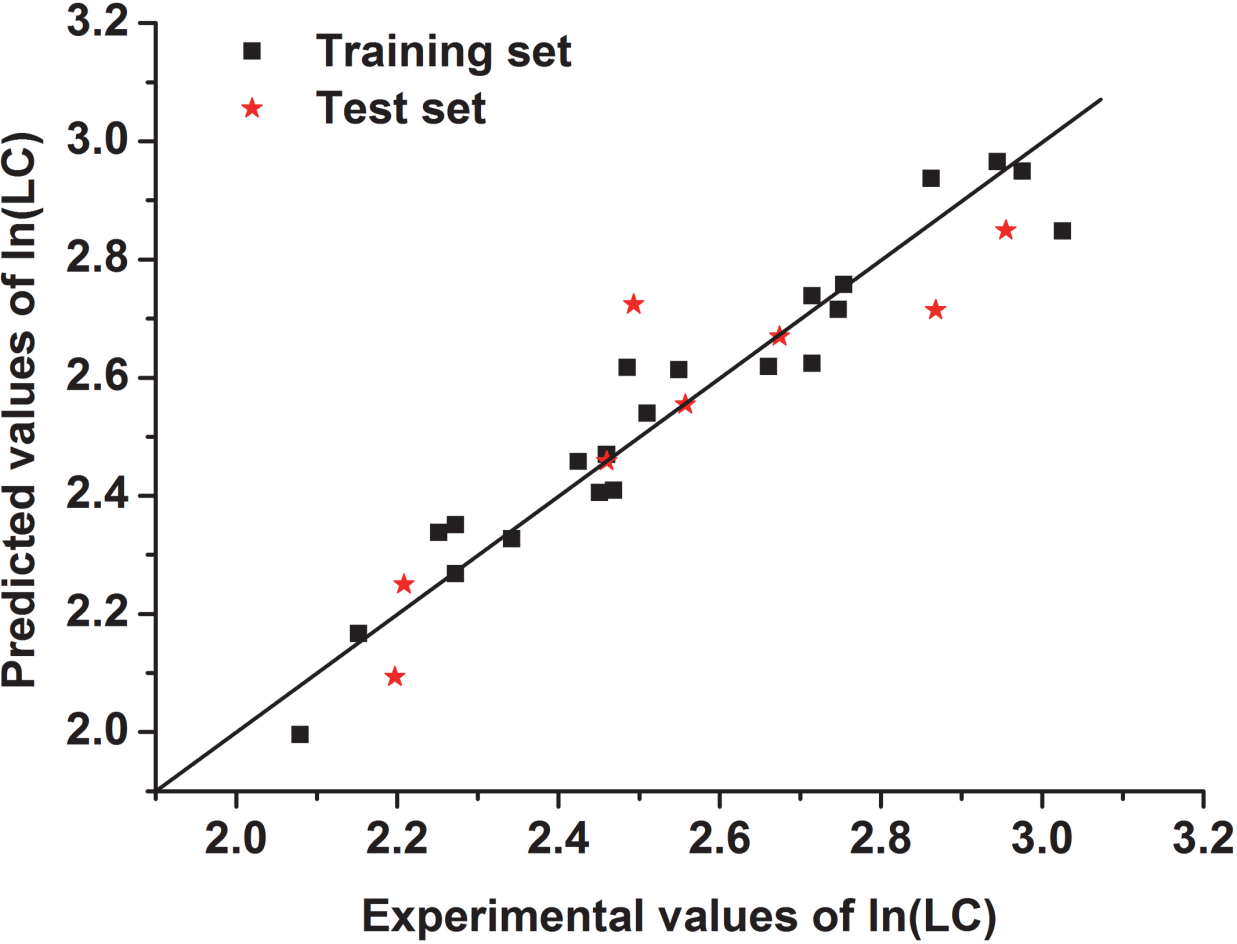
The linear correlation diagram between the predicted values and experimental values of ln(LC) for the training and test sets used for Model 1.

As seen in S6 Table, Model 1 shows the minimal residuals, possessing the optimal prediction ability. With regard to the optimization model, all of the predicted values of drug loading capacity of the samples in the training and test sets are close to their experimental values, as shown in S1 Table. Moreover, Fig. 4 shows that the sample points in the training and test sets are uniformly distributed along the line y = x, indicating that the residual errors of the predicted and experimental values are very low for Model 1. For example, the predicted value of No. 26 is 14.4, very similar to the experimental value 14.5, and the residual value (0.0040) is also much lower than 0.1.

S7 Table is the correlation matrix of the five descriptors in the optimization model. According to the previous research [51], all the correlation coefficients are less than 0.95, indicating the correlation analysis indicated that SSOV, SSA, EV TPE and IE are not highly correlated. Furthermore, if the multi-collinearity was present, in order to avoid its effects, probability (*p*) values of each coefficient are used to check whether multi-collinearity is affecting a correlation. It is generally accepted if the *p*-value is less than 0.05 [52]. Table 2 shows that all the p-values of five descriptors in Model 1 are very low (*p* ≤ 0.005), showing that multi-collinearity could not affect the correlation here.

**Table 2:** Different parameters of the GFA-MLR Model 1.

Descriptors	Unstandardized coefficients		95% Confidence interval of B		Standardized coefficient	*p*-value <
	B	std. error	lower limit	upper limit		
Intercept	2.548	0.017	2.512	2.584	—	0.005
SSOV	−1.953	0.143	−2.256	−1.650	−7.601	0.005
SSA	1.101	0.096	0.898	1.305	3.966	0.005
EV	0.339	0.034	0.266	0.412	1.162	0.005
TPE	0.320	0.034	0.248	0.392	1.339	0.005
IE	0.525	0.053	0.413	0.636	2.138	0.005

Based on the above analysis, the 5-parameter version of Model 1 was selected as the optimization model, as shown in Table 2. The equation for the optimization model is as following:

$$\begin{array}{l} \ln\left(LC\right)=-1.953\left[SSOV\right]+1.101\left[SSA\right]+0.339\left[EV\right]+0.320\left[TPE\right]+0.525\left[IE\right]+2.548\\ n=22;R^{2}=0.934;R^{2}{}_{cv}=0.888;RMSE=0.0675;F=45.31;R^{2}{}_{pred}=0.811;\bar{r_{m(test)}^{2}}=0.7500 \end{array}$$ (7)

SSOV: solvent surface occupied volume; SSA: solvent surface area; EV: ellipsoidal volume; TPE: total potential energy; IE: inversion energy. If the values of the five descriptors could be work out, the predicted LC could be given *via* the above equation. The values of five descriptors in training and test set are shown in S8 Table.

### Applicability domain of the optimization model

**Fig 5:**
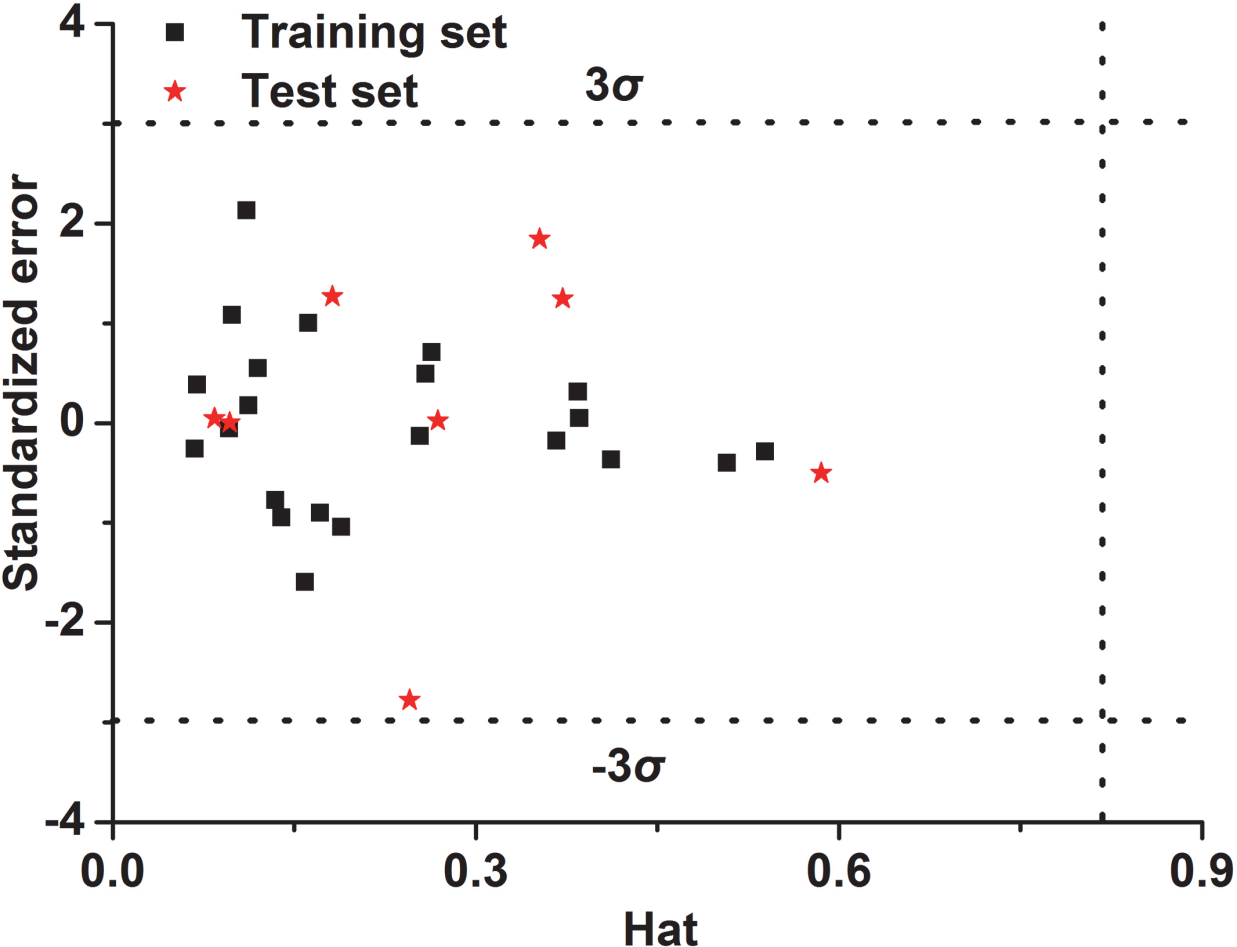
Williams plot of the optimization model.

Fig. 5 shows a Williams plot of the optimization model; this plot was developed using a leverage method to define the applicability domain of the model. All data points fall within the safety zone, except for one point, which is close to the bottom warning line but remains in the reasonable area. And the residual errors of the training and test sets are relatively smaller. Thus, the model was able to accurately predict the results.

### Descriptor interpretation and mechanism analysis

By interpreting the descriptors in the optimization model, it is possible to gain some insight into the factors that are likely to govern the ln(LC) of these polymeric micelles. The relative importance of the descriptors was determined based on their standardized regression coefficients. Table 2 shows that the most important descriptor is the SSOV, which is the solvent surface occupied volume of the polymers. This descriptor is mainly correlated with the hydrophilicity of a polymer molecule in aqueous solution. The SSA is a solvent surface area descriptor of the polymers and describes the hydrophobicity of polymer molecules in aqueous solution. EV is the ellipsoidal volume descriptor, which describes the volume of the ellipsoid of inertia, which is derived from the inertia tensor of the system. This descriptor is mainly correlated with the rigidity (indirectly reflecting the hydrophobicity) of polymer molecules in aqueous solution. IE is the inversion energy descriptor, which describes the energy required to transform a molecule from one spatial form (or invertomer) to another. This descriptor is a good indicator of the nature of the bonding between the atoms of a polymer.

**Table 3:** The correlation coefficient between the different segments and the five descriptors.

Segment	*SSOV*	*SSA*	*EV*	*TPE*	*IE*
PCL	0.55	0.31	0.60	0.54	0.72
PDEA	0.79	0.59	0.63	0.52	0.72
PPEGMA	−0.21	−0.03	−0.51	−0.01	−0.33
PCL+PDEA	0.79	0.52	0.74	0.63	0.86

Table 3 presents the relationship between the five descriptors and the three monomers. As shown in the table, positive correlations exist between PCL or PDEA and the five descriptors, whereas negative correlations exist between PPEGMA and the corresponding parameters. The correlation coefficients for the former group are slightly higher than those of monomers. An unknown synergistic effect might occur between the two hydrophobic blocks (PCL and PDEA).

**Table 4:** The contributions of the tested monomers to the five descriptors.

	*SSOV*	*SSA*	*EV*	*TPE*	*IE*
C_CL_	1.36	1.52	0.29	0.85	10
C_DEA_	1.27	1.30	0.39	1.28	114
C_PEGMA_	1	1	1	1	1

Table 4 shows the contributions of different segments to the descriptor values. The calculated values are based on the data presented in S9 Table using the PPEGMA monomer as a reference. For example, the contribution to *SSOV* caused by the DEAEMA monomer is calculated as follows: C_DEA_ = (705.01/1583.56)/(187.28/533.45) = 1.27. With regard to the descriptor *SSOV*, different contributions resulted from the three monomers in the order C_CL_ > C_DEA_ > C_PEGMA_.

As shown in Table 4, the hydrophobic segments (CL and DEA) in the polymer exhibited enhanced SSOV, resulting in a higher value of *SSOV*. A larger micellar core might be induced by segments that are more hydrophobic, thus providing a larger surface area for solvent and resulting in higher value of *SSOV*. Conversely, more hydrophilic segments (PEGMA) yield lower values of *SSOV*, possibly because the PEGMA segment distributed into the surface of the micelle. The same is true for *SSA*. The effect of the PEGMA segment on EV is greater than those of the CL and DEA segments. However, the relationship between hydrophilic segments and *EV* is negative, as shown in Table 3. The pH-sensitive DEA segment is the main factor for the descriptor *TPE*, and this segment exerts a positive affect. The DEA segment is also the largest contributor to the descriptor *IE* and provides a much greater contribution (up to 114-fold) than those of the CL (10-fold) and PEGMA (1-fold) segments. Equation (7) and S10 Table demonstrate that the descriptors *SSOV*, *SSA* and *IE* are the main factors in the optimization model. Because the contributions of the three descriptors are lower than that of *EV*, the coefficients of the three descriptors are much higher than that of *EV*; *IE* shows the most dramatic change among the five descriptors. The equation describing the model shows that the drug loading capacity can be enhanced by reducing *SSOV* and increasing the remaining four descriptors (*SSA*, *EV*, *TPE* and *IE*). According to the above analysis, although increases in CL and DEA enhance the values of *SSOV* (reducing LC) and the other four descriptors (increasing LC), the overall effect is to increase LC. An increase in PEGMA decreases the value of *SSOV* (increasing LC) but decreases the other descriptors, reducing the LC overall. In other words, to enhance the drug loading capacity of the micelles, the number of hydrophobic segments (DEA and CL) should be increased and the number of hydrophilic segments (PEGMA) should be decreased; this conclusion is consistent with the experimental observations.

Thus, the amounts of CL and DEA positively and significantly affect the drug loading capacity. The hydrophobic CL and DEA units are located in the core of the spherical micelle and provide sufficient space to accommodate the anticancer drug DOX in a synergistic manner. Decreasing the number of PEGMA units is also important for increasing the drug-loading capacity.

## Conclusions

This work addresses the QSPR between a series of amphiphilic four/six-arm star polymer structures and their micelle drug-loading capacities. Our study developed and evaluated ten models based on the GFA, the LOF function, MLR and LOO-cross-validation methods. The optimal descriptor number of the model was determined to be five based on the statistical results of the LOO-cross-validated correlation coefficient (*R* ^*2*^_*cv*_) and the square of the correlation coefficient (*R* ^*2*^). By analyzing the internal and external validation parameters of all models, the QSPR model was confirmed as optimal and to possess good fitting ability, good predictive ability and high stability. The influence of polymer structure on micelle drug-loading capacity was also analyzed. This study provides an effective approach for the design and synthesis of new star polymers with desired drug-loading capacities.

## Supporting Information

S1 Table The experimental and predicted values of drug-loading capacity (LC) of polymeric micelles in the training and test sets. (DOC)

S2 Table The 52 descriptors used in the QSPR analysis. (DOC)

S3 Table The 36 candidate descriptors used in the QSPR analysis. (DOC)

S4 Table The data of 36 candidate descriptors used in the QSPR analysis (the descriptor data is standardized according to the Mean/SD method). (XLS)

S5 TableEquations relating to $\bar{r_{m}^{2}}$ and $\Delta r_{m}^{2}$.(DOC)

S6 Table The absolute residuals of ten models. (DOC)

S7 Table The Correlation Matrix of the optimization model. (DOC)

S8 Table The values of five descriptors in the training and test sets (the descriptor data is standardized according to the Mean/SD method). (DOC)

S9 Table The contributions of five descriptors according to three units. (DOC)

S10 Table The values of five descriptors in the training and test sets. (DOC)
