# Supplementary material for: Quantitative Structure-Property Relationship (QSPR) Modeling of Drug-Loaded Polymeric Micelles via Genetic Function Approximation
Source: PLoS One. 2015 Mar 17;10(3):e0119575. doi: 10.1371/journal.pone.0119575 (PMC4364361; doi:10.1371/journal.pone.0119575)
Supplement: S1 Table — (DOC) [file pone.0119575.s001.doc]

**S1 Table. The experimental and predicted values of drug-loading capacity (LC) of polymeric micelles in the training and test sets.**

| **No** | **Micelles** | **Polymers** | **LC** | **LC^#^** | **ln(LC)** | **ln(LC^#^)** |
| --- | --- | --- | --- | --- | --- | --- |
| **Training set** | | | | | | |
| 1 | 4ASP2-M-a | (PCL_24_)_2_(PDEA_37_-*b*-PPEGMA_15_)_2_ | 11.6 | 11.1 | 2.4510 | 2.4051 |
| 2 | 4ASP4-M-a | (PCL_38_)_2_(PDEA_17_-*b*-PPEGMA_9_)_2_ | 15.7 | 15.8 | 2.7537 | 2.7579 |
| 3 | 4ASP6-M-a | (PCL_32_)_2_(PDEA_20_-*b*-PPEGMA_19_)_2_ | 9.7 | 10.5 | 2.2721 | 2.3507 |
| 4 | 4ASP1-M-b | (PCL_24_)_2_(PDEA_16_-*b*-PPEGMA_19_)_2_ | 11.8 | 11.1 | 2.4681 | 2.4090 |
| 5 | 4ASP3-M-b | (PCL_38_)_2_(PDEA_26_-*b*-PPEGMA_11_)_2_ | 17.5 | 18.9 | 2.8622 | 2.9372 |
| 6 | 4ASP4-M-b | (PCL_38_)_2_(PDEA_17_-*b*-PPEGMA_9_)_2_ | 19.0 | 19.4 | 2.9444 | 2.9655 |
| 7 | 4ASP5-M-b | (PCL_32_)_2_(PDEA_25_-*b*-PPEGMA_22_)_2_ | 12.3 | 12.7 | 2.5096 | 2.5397 |
| 8 | 4ASP6-M-b | (PCL_32_)_2_(PDEA_20_-*b*-PPEGMA_19_)_2_ | 15.1 | 13.8 | 2.7147 | 2.6245 |
| 9 | 6ASP1-M-a | (PCL_16_)_3_(PDEA_14_-*b*-PPEGMA_10_)_3_ | 9.5 | 10.4 | 2.2513 | 2.3380 |
| 10 | 6ASP3-M-a | (PCL_33_)_3_(PDEA_12_-*b*-PPEGMA_10_)_3_ | 14.3 | 13.7 | 2.6603 | 2.6191 |
| 11 | 6ASP4-M-a | (PCL_33_)_3_(PDEA_21_-*b*-PPEGMA_11_)_3_ | 15.1 | 15.5 | 2.7147 | 2.7384 |
| 12 | 6ASP1-M-b | (PCL_16_)_3_(PDEA_14_-*b*-PPEGMA_10_)_3_ | 12.8 | 13.7 | 2.5494 | 2.6138 |
| 13 | 6ASP2-M-b | (PCL_25_)_3_(PDEA_15_-*b*-PPEGMA_12_)_3_ | 15.6 | 15.1 | 2.7473 | 2.7151 |
| 14 | 6ASP4-M-b | (PCL_33_)_3_(PDEA_21_-*b*-PPEGMA_11_)_3_ | 19.6 | 19.1 | 2.9755 | 2.9492 |
| 15 | 4ASP1-H-a | (PCL_22_-b-PDEA_25_-*b*-PPEGMA_5_)_4_ | 8.0 | 7.4 | 2.0794 | 1.9957 |
| 16 | 4ASP2-H-a | (PCL_35_-b-PDEA_15_-*b*-PPEGMA_5_)_4_ | 12.0 | 13.7 | 2.4849 | 2.6176 |
| 17 | 4ASP3-H-a | (PCL_35_-b-PDEA_26_-*b*-PPEGMA_4_)_4_ | 11.3 | 11.7 | 2.4248 | 2.4578 |
| 18 | 4ASP1-H-b | (PCL_22_-b-PDEA_25_-*b*-PPEGMA_5_)_4_ | 10.4 | 10.2 | 2.3418 | 2.3272 |
| 19 | 4ASP2-H-b | (PCL_35_-b-PDEA_15_-*b*-PPEGMA_5_)_4_ | 20.6 | 17.3 | 3.0253 | 2.8480 |
| 20 | 6ASP1-H-a | (PCL_18_-b-PDEA_9_-*b*-PPEGMA_4_)_6_ | 9.7 | 9.7 | 2.2721 | 2.2678 |
| 21 | 6ASP2-H-a | (PCL_18_-b-PDEA_15_-*b*-PPEGMA_4_)_6_ | 8.6 | 8.7 | 2.1518 | 2.1665 |
| 22 | 6ASP2-H-b | (PCL_18_-b-PDEA_15_-*b*-PPEGMA_4_)_6_ | 11.7 | 11.8 | 2.4596 | 2.4702 |
| **Test set** | | | | | | |
| 23 | 4ASP1-M-a | (PCL_24_)_2_(PDEA_16_-*b*-PPEGMA_19_)_2_ | 9.0 | 8.1 | 2.1972 | 2.0935 |
| 24 | 4ASP3-M-a | (PCL_38_)_2_(PDEA_26_-*b*-PPEGMA_11_)_2_ | 12.1 | 15.2 | 2.4932 | 2.7243 |
| 25 | 4ASP5-M-a | (PCL_32_)_2_(PDEA_25_-*b*-PPEGMA_22_)_2_ | 9.1 | 9.5 | 2.2083 | 2.2499 |
| 26 | 4ASP2-M-b | (PCL_24_)_2_(PDEA_37_-*b*-PPEGMA_15_)_2_ | 14.5 | 14.4 | 2.6741 | 2.6702 |
| 27 | 6ASP2-M-a | (PCL_25_)_3_(PDEA_15_-*b*-PPEGMA_12_)_3_ | 11.7 | 11.7 | 2.4596 | 2.4591 |
| 28 | 6ASP3-M-b | (PCL_33_)_3_(PDEA_12_-*b*-PPEGMA_10_)_3_ | 19.2 | 17.3 | 2.9549 | 2.8493 |
| 29 | 4ASP3-H-b | (PCL_35_-b-PDEA_26_-*b*-PPEGMA_4_)_4_ | 17.6 | 15.1 | 2.8679 | 2.7145 |
| 30 | 6ASP1-H-b | (PCL_18_-b-PDEA_9_-*b*-PPEGMA_4_)_6_ | 12.9 | 12.9 | 2.5572 | 2.5551 |

: The “a” denotes that doxorubicin/polymer ratio is 10 mg/40 mg; : The “b” denotes that doxorubicin/polymer ratio is 20 mg / 40 mg; : The “LC” is the experimental value, The “ln(LC)” is natural logarithm of LC value; : The “LC^#^” is predicted value.
