# Supplementary material for: Quantitative Structure-Property Relationship (QSPR) Modeling of Drug-Loaded Polymeric Micelles via Genetic Function Approximation
Source: PLoS One. 2015 Mar 17;10(3):e0119575. doi: 10.1371/journal.pone.0119575 (PMC4364361; doi:10.1371/journal.pone.0119575)
Supplement: S2 Table — (DOC) [file pone.0119575.s002.doc]

**S2 Table.** The 52 descriptors used in the QSPR analysis.

| **Category of**  **descriptors** | **Descriptors** |
| --- | --- |
| Spatial Descriptors | Dipole moment (magnitude), Dipole moment X, Dipole moment Y, Dipole moment Z, Shadow area: XY plane, Shadow area: YZ plane, Shadow area: ZX plane, Shadow area fraction: XY plane, Shadow area fraction: YZ plane, Shadow area fraction: ZX plane, Shadow length: LX, Shadow length: LY, Shadow length: LZ, Shadow ratio, Ellipsoidal volume, Radius of gyration, Principal moments of inertia (magnitude), Principal moment of inertia X, Principal moment of inertia Y, Principal moment of inertia Z, Molecular density, Molecular volume (vdW volume), Molecular area (vdW area) |
| Atom Volumes and Surfaces | Solvent surface occupied volume, Solvent surface area, Connolly surface area, Connolly surface occupied volume |
| Atomistic Descriptors | Element count, Atom count, Total molecular mass |
| Fragment Counts | Ethyl, Methyl, Hydroxy, Methoxy |
| Thermodynamic | Total potential energy, Bond energy, Angle energy, Torsion energy, Inversion energy, van der Waals energy, Electrostatic energy, Non-bond energy, Log *P*, Refractivity, Polarizability |
| Topological | Cluster count, Molecular topological index, Num rotatable bonds, Polar surface area, Shape attribute, Sum of degrees, Sum of valence degrees, Wiener index |
