# Supplementary material for: Quantitative Structure-Property Relationship (QSPR) Modeling of Drug-Loaded Polymeric Micelles via Genetic Function Approximation
Source: PLoS One. 2015 Mar 17;10(3):e0119575. doi: 10.1371/journal.pone.0119575 (PMC4364361; doi:10.1371/journal.pone.0119575)
Supplement: S3 Table — (DOC) [file pone.0119575.s003.doc]

**S3 Table.** The 36 candidate descriptors used in the QSPR analysis.

| **Category of**  **descriptors** | **Descriptors** |
| --- | --- |
| **Spatial descriptors** | Dipole moment, Dipole moment X, Dipole moment Y, Dipole moment Z, Shadow area: XY plane, Shadow area: YZ plane, Shadow area: ZX plane, Shadow area fraction: XY plane, Shadow area fraction: YZ plane, Shadow area fraction: ZX plane, Shadow length: LX, Shadow length: LY, Shadow length: LZ, Shadow ratio, Ellipsoidal volume, Radius of gyration, Principal moment of inertia X, Principal moment of inertia Z, Molecular density |
| **Atom volumes and surfaces** | Solvent surface occupied volume, Solvent surface area |
| **Fragment counts** | Ethyl, Methyl, Hydroxy, Methoxy |
| **Thermodynamic** | Total potential energy, Angle energy, Torsion energy, Inversion energy, van der Waals energy, Electrostatic energy, Non-bond energy, Log *P*, Refractivity |
| **Topological** | Num rotatable bonds, Wiener index |
