# Supplementary material for: Quantitative Structure-Property Relationship (QSPR) Modeling of Drug-Loaded Polymeric Micelles via Genetic Function Approximation
Source: PLoS One. 2015 Mar 17;10(3):e0119575. doi: 10.1371/journal.pone.0119575 (PMC4364361; doi:10.1371/journal.pone.0119575)
Supplement: S5 Table — (DOC) [file pone.0119575.s005.doc]

**S5 Table.** Equations relating to $\overline{r_{m}^{2}}$ and $\Delta r_{m}^{2}$.

| **Equations** | **Note** |
| --- | --- |
| $\begin{matrix} r^{2}=\frac{\left[\sum\left(Y_{obs}-\bar{Y}_{obs}\right)\left(Y_{pred}-\bar{Y}_{pred}\right)\right]^{2}}{\sum\left(Y_{pred}-\bar{Y}_{pred}\right)^{2}\times\sum\left(Y_{obs}-\bar{Y}_{obs}\right)^{2}} \\ =\frac{\left[\sum\left(Y_{pred}-\bar{Y}_{pred}\right)\left(Y_{obs}-\bar{Y}_{obs}\right)\right]^{2}}{\sum\left(Y_{obs}-\bar{Y}_{obs}\right)^{2}\times\sum\left(Y_{pred}-\bar{Y}_{pred}\right)^{2}} \end{matrix}$ | $Y_{obs}$: the experimental property of the training set sample;  $\bar{Y}_{obs}$: the average experimental property of the training set sample;  $Y_{pred}$: the predicted property of the training set sample;  $\bar{Y}_{pred}$: the average predicted property of the training set sample. |
| $r_{0}^{2}=1-\frac{\sum\left(Y_{obs}-k\times Y_{pred}\right)^{2}}{\sum\left(Y_{obs}-\bar{Y}_{obs}\right)^{2}}$ |  |
| $r_{0}^{'2}=1-\frac{\sum\left(Y_{pred}-k^{'}\times Y_{obs}\right)^{2}}{\sum\left(Y_{pred}-\bar{Y}_{pred}\right)^{2}}$ |  |
| $k=\frac{\sum\left(Y_{obs}\times Y_{pred}\right)}{\sum\left(Y_{pred}\right)^{2}}$ | The parameters k and $k^{'}$indicate the slopes in the former and later cases respectively. |
| $k^{'}=\frac{\sum\left(Y_{obs}\times Y_{pred}\right)}{\sum\left(Y_{obs}\right)^{2}}$ |  |
| $r_{m}^{2}=r^{2}\times\left(1-\sqrt{r^{2}-r_{0}^{2}}\right)$ | $r_{m}^{2}$is calculated based on the correlations between observed and predicted values with (r^2^) and without (r_0_^2^) intercept for the least squares regression lines. |
| $r_{m}^{'2}=r^{2}\times\left(1-\sqrt{r^{2}-r_{0}^{'2}}\right)$ |  |
| $\overline{r_{m}^{2}}=\left(r_{m}^{2}-r_{m}^{'2}\right)/2$ |  |
| $\Delta r_{m}^{2}=\left\vert r_{m}^{2}-r_{m}^{'2}\right\vert$ |  |
