# Supplementary material for: Quantitative Structure-Property Relationship (QSPR) Modeling of Drug-Loaded Polymeric Micelles via Genetic Function Approximation
Source: PLoS One. 2015 Mar 17;10(3):e0119575. doi: 10.1371/journal.pone.0119575 (PMC4364361; doi:10.1371/journal.pone.0119575)
Supplement: S6 Table — (DOC) [file pone.0119575.s006.doc]

**S6 Table. The absolute residuals of ten models**

| **No** | **log(LC)** | **Model 1** | **Model 2** | **Model 3** | **Model 4** | **Model 5** | **Model 6** | **Model 7** | **Model 8** | **Model 9** | **Model 10** |
| --- | --- | --- | --- | --- | --- | --- | --- | --- | --- | --- | --- |
| **Training set** | | | | | | | | | | | |
| **1** | 2.4510 | 0.0459 | 0.0603 | 0.0179 | 0.0747 | 0.0846 | 0.0866 | 0.0409 | 0.0955 | 0.0498 | 0.0260 |
| **2** | 2.7537 | 0.0042 | 0.0216 | 0.0066 | 0.0089 | 0.0259 | 0.0097 | 0.0037 | 0.0266 | 0.0038 | 0.0396 |
| **3** | 2.2721 | 0.0786 | 0.0695 | 0.0688 | 0.1024 | 0.0887 | 0.1076 | 0.0884 | 0.0920 | 0.0906 | 0.1081 |
| **4** | 2.4681 | 0.0591 | 0.0486 | 0.0764 | 0.0378 | 0.0298 | 0.0312 | 0.0589 | 0.0244 | 0.0550 | 0.1276 |
| **5** | 2.8622 | 0.0750 | 0.0927 | 0.1156 | 0.0683 | 0.0869 | 0.0645 | 0.1113 | 0.0836 | 0.1087 | 0.0016 |
| **6** | 2.9444 | 0.0211 | 0.0457 | 0.0307 | 0.0220 | 0.0469 | 0.0226 | 0.0314 | 0.0479 | 0.0321 | 0.0270 |
| **7** | 2.5096 | 0.0301 | 0.0254 | 0.0602 | 0.0135 | 0.0121 | 0.0101 | 0.0483 | 0.0098 | 0.0473 | 0.0199 |
| **8** | 2.7147 | 0.0902 | 0.0878 | 0.0798 | 0.0733 | 0.0741 | 0.0691 | 0.0651 | 0.0709 | 0.0626 | 0.0893 |
| **9** | 2.2513 | 0.0867 | 0.0684 | 0.0707 | 0.0867 | 0.0683 | 0.0875 | 0.0704 | 0.0689 | 0.0709 | 0.0344 |
| **10** | 2.6603 | 0.0412 | 0.0682 | 0.0782 | 0.0988 | 0.1176 | 0.1214 | 0.1300 | 0.1380 | 0.1478 | 0.1033 |
| **11** | 2.7147 | 0.0237 | 0.0301 | 0.0186 | 0.0244 | 0.0302 | 0.0221 | 0.0187 | 0.0281 | 0.0172 | 0.0420 |
| **12** | 2.5494 | 0.0644 | 0.0591 | 0.0696 | 0.0614 | 0.0567 | 0.0620 | 0.0677 | 0.0576 | 0.0688 | 0.0032 |
| **13** | 2.7473 | 0.0322 | 0.0660 | 0.0540 | 0.0029 | 0.0445 | 0.0055 | 0.0308 | 0.0384 | 0.0255 | 0.1832 |
| **14** | 2.9755 | 0.0263 | 0.0110 | 0.0120 | 0.0288 | 0.0132 | 0.0307 | 0.0136 | 0.0146 | 0.0141 | 0.0348 |
| **15** | 2.0794 | 0.0837 | 0.0506 | 0.0600 | 0.0900 | 0.0524 | 0.0907 | 0.0629 | 0.0521 | 0.0629 | 0.0194 |
| **16** | 2.4849 | 0.1327 | 0.0896 | 0.0660 | 0.1215 | 0.0778 | 0.1185 | 0.0525 | 0.0747 | 0.0496 | 0.1916 |
| **17** | 2.4248 | 0.0330 | 0.0566 | 0.0427 | 0.0633 | 0.0824 | 0.0720 | 0.0686 | 0.0896 | 0.0741 | 0.0632 |
| **18** | 2.3418 | 0.0146 | 0.0231 | 0.0239 | 0.0229 | 0.0192 | 0.0236 | 0.0197 | 0.0199 | 0.0204 | 0.0154 |
| **19** | 3.0253 | 0.1773 | 0.2034 | 0.2145 | 0.1897 | 0.2156 | 0.1923 | 0.2275 | 0.2178 | 0.2293 | 0.1517 |
| **20** | 2.2721 | 0.0043 | 0.0326 | 0.0407 | 0.0102 | 0.0376 | 0.0068 | 0.0467 | 0.0339 | 0.0431 | 0.1023 |
| **21** | 2.1518 | 0.0147 | 0.0209 | 0.0307 | 0.0334 | 0.0373 | 0.0409 | 0.0479 | 0.0439 | 0.0535 | 0.0809 |
| **22** | 2.4596 | 0.0106 | 0.0259 | 0.0426 | 0.0232 | 0.0372 | 0.0294 | 0.0553 | 0.0431 | 0.0607 | 0.0933 |
| **Test set** | | | | | | | | | | | |
| **23** | 2.1972 | 0.1037 | 0.1035 | 0.1471 | 0.0745 | 0.0777 | 0.0665 | 0.1240 | 0.0720 | 0.1200 | 0.1475 |
| **24** | 2.4932 | 0.2311 | 0.2397 | 0.2567 | 0.2267 | 0.2363 | 0.2225 | 0.2537 | 0.2315 | 0.2500 | 0.1716 |
| **25** | 2.2083 | 0.0417 | 0.0222 | 0.0532 | 0.0259 | 0.0120 | 0.0227 | 0.0413 | 0.0071 | 0.0403 | 0.0099 |
| **26** | 2.6741 | 0.0040 | 0.0066 | 0.0373 | 0.0309 | 0.0283 | 0.0406 | 0.0165 | 0.0376 | 0.0101 | 0.0324 |
| **27** | 2.4596 | 0.0005 | 0.0535 | 0.0496 | 0.0382 | 0.0240 | 0.0487 | 0.0197 | 0.0177 | 0.0139 | 0.1525 |
| **28** | 2.9549 | 0.1056 | 0.1185 | 0.1184 | 0.1568 | 0.1617 | 0.1756 | 0.1634 | 0.1787 | 0.1774 | 0.1814 |
| **29** | 2.8679 | 0.1534 | 0.1252 | 0.1285 | 0.1306 | 0.1033 | 0.1226 | 0.1083 | 0.0985 | 0.1021 | 0.1522 |
| **30** | 2.5572 | 0.0022 | 0.0161 | 0.0144 | 0.0100 | 0.0221 | 0.0071 | 0.0210 | 0.0189 | 0.0171 | 0.0633 |
