# Supplementary material for: Quantitative Structure-Property Relationship (QSPR) Modeling of Drug-Loaded Polymeric Micelles via Genetic Function Approximation
Source: PLoS One. 2015 Mar 17;10(3):e0119575. doi: 10.1371/journal.pone.0119575 (PMC4364361; doi:10.1371/journal.pone.0119575)
Supplement: S7 Table — (DOC) [file pone.0119575.s007.doc]

**S7 Table.** The Correlation Matrix of the optimization model

|  | **SSOV** | **SSA** | **EV** | **TPE** | **IE** |
| --- | --- | --- | --- | --- | --- |
| **SSOV** | 1.0000 |  |  |  |  |
| **SSA** | 0.9220 | 1.0000 |  |  |  |
| **EV** | 0.6993 | 0.7460 | 1.0000 |  |  |
| **TPE** | 0.7856 | 0.5888 | 0.3070 | 1.0000 |  |
| **IE** | 0.7819 | 0.5158 | 0.3236 | 0.8082 | 1.0000 |
