# Supplementary material for: Quantitative Structure-Property Relationship (QSPR) Modeling of Drug-Loaded Polymeric Micelles via Genetic Function Approximation
Source: PLoS One. 2015 Mar 17;10(3):e0119575. doi: 10.1371/journal.pone.0119575 (PMC4364361; doi:10.1371/journal.pone.0119575)
Supplement: S8 Table — (DOC) [file pone.0119575.s008.doc]

**S8 Table.** The values of five descriptors in the training and test sets (the descriptor data is standardized according to the Mean/SD method).

| **No** | **Polymer** | **SSOV** | **SSA** | **EV** | **TPE** | **IE** |
| --- | --- | --- | --- | --- | --- | --- |
| **Training set** | | | | | | |
| 1 | 4ASP2-M-1 | 0.6422 | 0.8500 | 0.1385 | -0.0542 | 0.2779 |
| 2 | 4ASP4-M-1 | -1.2029 | -0.9471 | -0.6915 | -1.2660 | -0.8695 |
| 3 | 4ASP6-M-1 | 0.4191 | 0.7853 | -0.1261 | -0.2658 | -0.2204 |
| 4 | 4ASP1-M-2 | -1.0424 | -0.4927 | -0.8440 | -1.2805 | -1.7842 |
| 5 | 4ASP3-M-2 | -1.1609 | -1.0258 | -0.6764 | -1.1283 | -0.3002 |
| 6 | 4ASP4-M-2 | -1.9658 | -1.7159 | -0.8169 | -1.7408 | -1.3295 |
| 7 | 4ASP5-M-2 | 0.1984 | 0.5284 | 1.7011 | -0.5233 | -1.1654 |
| 8 | 4ASP6-M-2 | -0.6075 | -0.2650 | -0.3434 | -0.9031 | -0.7859 |
| 9 | 6ASP1-M-1 | -0.6847 | -0.5037 | -0.8201 | -0.4846 | -1.0657 |
| 10 | 6ASP3-M-1 | 0.3400 | 0.4178 | -0.5834 | 0.1819 | 0.7907 |
| 11 | 6ASP4-M-1 | 1.3835 | 1.3125 | 0.0668 | 2.8216 | 0.9943 |
| 12 | 6ASP1-M-2 | -1.5319 | -1.3446 | -0.9246 | -1.0864 | -1.4938 |
| 13 | 6ASP2-M-2 | -0.3224 | -0.1868 | -0.3415 | -0.0284 | -0.2506 |
| 14 | 6ASP4-M-2 | 0.2003 | 0.1765 | -0.1818 | 1.6827 | 0.2314 |
| 15 | 4ASP1-H-1 | 1.2596 | 0.9854 | 0.5466 | 0.8069 | 0.7231 |
| 16 | 4ASP2-H-1 | 0.9280 | 0.6909 | 1.2151 | 0.8124 | 0.8567 |
| 17 | 4ASP3-H-1 | 2.3771 | 2.2077 | 2.5773 | 0.9552 | 1.7955 |
| 18 | 4ASP1-H-2 | 0.0965 | -0.0975 | 0.2200 | -0.0047 | 0.0043 |
| 19 | 4ASP2-H-2 | -0.1813 | -0.3441 | 0.7799 | -0.0001 | 0.1162 |
| 20 | 6ASP1-H-1 | -0.4199 | -1.2722 | -0.6814 | 0.7227 | 0.5738 |
| 21 | 6ASP2-H-1 | 0.9833 | 0.0041 | -0.6445 | 1.6097 | 2.3598 |
| 22 | 6ASP2-H-2 | -0.1350 | -0.9193 | -0.7775 | 0.6676 | 1.3750 |
| **Test set** | | | | | | |
| 23 | 4ASP1-M-1 | -0.1003 | 0.5135 | -0.7239 | -0.7165 | -1.4124 |
| 24 | 4ASP3-M-1 | -0.2417 | -0.1230 | -0.5238 | -0.5347 | 0.3595 |
| 25 | 4ASP5-M-1 | 1.3813 | 1.7326 | 2.3150 | 0.1877 | -0.6735 |
| 26 | 4ASP2-M-2 | -0.4206 | -0.2108 | -0.1217 | -0.7259 | -0.3685 |
| 27 | 6ASP2-M-1 | 0.7595 | 0.8787 | -0.1239 | 0.7786 | 0.4187 |
| 28 | 6ASP3-M-2 | -0.6737 | -0.5728 | -0.7263 | -0.5282 | 0.0609 |
| 29 | 4ASP3-H-2 | 1.0324 | 0.9262 | 1.9207 | 0.1195 | 0.9024 |
| 30 | 6ASP1-H-2 | -1.3101 | -1.9882 | -0.8084 | -0.0752 | -0.1207 |
