# Supplementary material for: Quantitative Structure-Property Relationship (QSPR) Modeling of Drug-Loaded Polymeric Micelles via Genetic Function Approximation
Source: PLoS One. 2015 Mar 17;10(3):e0119575. doi: 10.1371/journal.pone.0119575 (PMC4364361; doi:10.1371/journal.pone.0119575)
Supplement: S9 Table — (DOC) [file pone.0119575.s009.doc]

**S9 Table.** The contributions of five descriptors according to three units.

| Monomer | M/g•mol^-1^ | SSOV/Å^3^ | SSA/Å^2^ | EV/Å^3^ | TPE/kcal•mol^-1^ | IE/ kcal•mol^-1^ |
| --- | --- | --- | --- | --- | --- | --- |
| CL | 116.16 | 471.92 | 327.40 | 399.96 | 9.60 | 0.001043 |
| DEA | 187.28 | 705.01 | 452.42 | 848.30 | 35.08 | 0.019161 |
| PEGMA | 533.45 | 1583.56 | 987.41 | 6420.69 | 77.89 | 4.6806E+04 |

The values of five descriptors are calculated using Materials Studio 5.0 after geometric structures of monomer molecules were optimized.
