# Supplementary material for: Quantitative Structure-Property Relationship (QSPR) Modeling of Drug-Loaded Polymeric Micelles via Genetic Function Approximation
Source: PLoS One. 2015 Mar 17;10(3):e0119575. doi: 10.1371/journal.pone.0119575 (PMC4364361; doi:10.1371/journal.pone.0119575)
Supplement: S10 Table — (DOC) [file pone.0119575.s010.doc]

**S10 Table.** The values of five descriptors in the training and test sets.

| **No** | **Polymer** | **SSOV**  **/10^4^Å^3^** | **SSA**  **/10^4^Å^2^** | **EV**  **/10^6^Å^3^** | **TPE**  **/10^4^ kcal•mol^-1^** | **IE**  **/10 kcal•mol^-1^** |
| --- | --- | --- | --- | --- | --- | --- |
| **Training set** | | | | | | |
| 1 | 4ASP2-M-1 | 7.1694 | 3.1700 | 1.7031 | 1.6731 | 4.1797 |
| 2 | 4ASP4-M-1 | 5.1849 | 2.3181 | 0.82113 | 1.1984 | 2.9777 |
| 3 | 4ASP6-M-1 | 6.9295 | 3.1394 | 1.4219 | 1.5902 | 3.6577 |
| 4 | 4ASP1-M-2 | 5.3574 | 2.5336 | 0.65913 | 1.1927 | 2.0195 |
| 5 | 4ASP3-M-2 | 5.2300 | 2.2809 | 0.83718 | 1.2524 | 3.5741 |
| 6 | 4ASP4-M-2 | 4.3642 | 1.9537 | 0.68792 | 1.0124 | 2.4959 |
| 7 | 4ASP5-M-2 | 6.6921 | 3.0176 | 3.3635 | 1.4893 | 2.6678 |
| 8 | 4ASP6-M-2 | 5.8253 | 2.6415 | 1.1911 | 1.3406 | 3.0653 |
| 9 | 6ASP1-M-1 | 5.7422 | 2.5283 | 0.68449 | 1.5045 | 2.7722 |
| 10 | 6ASP3-M-1 | 6.8444 | 2.9651 | 0.93604 | 1.7656 | 4.7168 |
| 11 | 6ASP4-M-1 | 7.9669 | 3.3893 | 1.6269 | 2.7996 | 4.9301 |
| 12 | 6ASP1-M-2 | 4.8310 | 2.1298 | 0.57348 | 1.2688 | 2.3238 |
| 13 | 6ASP2-M-2 | 6.1320 | 2.6785 | 1.1931 | 1.6832 | 3.6260 |
| 14 | 6ASP4-M-2 | 6.6941 | 2.8508 | 1.3628 | 2.3534 | 4.1310 |
| 15 | 4ASP1-H-1 | 7.8336 | 3.2342 | 2.1367 | 2.0104 | 4.6460 |
| 16 | 4ASP2-H-1 | 7.4768 | 3.0946 | 2.8471 | 2.0125 | 4.7860 |
| 17 | 4ASP3-H-1 | 9.0356 | 3.8136 | 4.2946 | 2.0685 | 5.7694 |
| 18 | 4ASP1-H-2 | 6.5825 | 2.7209 | 1.7897 | 1.6925 | 3.8931 |
| 19 | 4ASP2-H-2 | 6.2837 | 2.6040 | 2.3847 | 1.6943 | 4.0103 |
| 20 | 6ASP1-H-1 | 6.0271 | 2.1640 | 0.83193 | 1.9774 | 4.4897 |
| 21 | 6ASP2-H-1 | 7.5363 | 2.7690 | 0.87108 | 2.3248 | 6.3605 |
| 22 | 6ASP2-H-2 | 6.3335 | 2.3313 | 0.72975 | 1.9558 | 5.3289 |
| **Test set** | | | | | | |
| 23 | 4ASP1-M-1 | 6.3709 | 3.0105 | 0.78675 | 1.4137 | 2.4090 |
| 24 | 4ASP3-M-1 | 6.2187 | 2.7088 | 0.99935 | 1.4849 | 4.2651 |
| 25 | 4ASP5-M-1 | 7.9645 | 3.5884 | 4.0159 | 1.7678 | 3.1830 |
| 26 | 4ASP2-M-2 | 6.0263 | 2.6672 | 1.4266 | 1.4100 | 3.5025 |
| 27 | 6ASP2-M-1 | 7.2956 | 3.1836 | 1.4243 | 1.9993 | 4.3272 |
| 28 | 6ASP3-M-2 | 5.7540 | 2.4956 | 0.78416 | 1.4874 | 3.9523 |
| 29 | 4ASP3-H-2 | 7.5892 | 3.2062 | 3.5969 | 1.7411 | 4.8339 |
| 30 | 6ASP1-H-2 | 5.0695 | 1.8246 | 0.69696 | 1.6649 | 3.7621 |
